# Supplementary material for: Protocol for development and validation of a prediction model for post-induction hypotension in elderly patients undergoing non-cardiac surgery: a prospective cohort study
Source: BMJ Open. 2023 Sep 21;13(9):e074181. doi: 10.1136/bmjopen-2023-074181 (PMC10514608; doi:10.1136/bmjopen-2023-074181)
Supplement: Supplementary data [file bmjopen-2023-074181supp002.pdf]

Case Report Form

**Preoperative potential predictors (15)**	
Age	
Gender	□Male □Female
BMI	
ASA physical status	□I □II □III
Hypertension	□Yes □No
Diabetes mellitus	□Yes □No
The age-adjusted Charlson Comorbidity Index (aCCI)	
Autonomic nervous system function	□Normal □Abnormal
Cardiac function	□Normal □Abnormal
NIBP in the ward	/ (MAP:)
HR in the ward	
The APAIS score (the first four items)	
The FRAIL scale score	□0 □1 □2
The volume of fluid infusion on the day of surgery	
Fasting duration on the day of surgery	
**Intraoperative potential predictors (8)**	
NIBP before induction	/ (MAP:)
HR before induction	
Dose of propofol	
Dose of etomidate	
Dose of ciprofol	
Dose of esketamine	
Dose of fentanyl	
Dose of sufentanil	

30% reduction in MAP from baseline threshold: ______________

**Outcome**				
**Time point**	**HR（bpm）**	**NIBP（mmHg）**	**MAP（mmHg）**	**PIH#**
Immediately after induction		/		□Yes □No
1min after induction		/		□Yes □No
2min after induction		/		□Yes □No
3min after induction		/		□Yes □No
4min after induction		/		□Yes □No
5min after induction		/		□Yes □No
6min after induction		/		□Yes □No
7min after induction		/		□Yes □No
8min after induction		/		□Yes □No
9min after induction		/		□Yes □No
10min after induction		/		□Yes □No
11min after induction		/		□Yes □No
12min after induction		/		□Yes □No
13min after induction		/		□Yes □No
14min after induction		/		□Yes □No
15min after induction		/		□Yes □No
Skin incision*		/		□Yes □No

* The first 15 minutes after anaesthesia induction or before skin incision (whichever occurred first)

# Hypotension is defined as a 30% reduction in MAP from baseline or MAP ≤65 mmHg.

The age-adjusted Charlson Comorbidity Index

Weight	Comorbid Condition
**1**	Myocardial infarction, congestive heart failure, peripheral vascular disease, cerebral vascular disease, dementia, chronic obstructive pulmonary disease, connective tissue disease, ulcer disease, mild liver disease, diabetes
**2**	Hemiplegia, moderate/severe renal disease, diabetes with end-organ damage, any tumor, leukemia/lymphoma
**3**	Moderate/severe liver disease
**6**	Metastatic solid tumor， AIDS
**1**	For each decade over age 40 years, up to 4 points
**Total**	
AIDS=acquired immune deficiency syndrome.	

The symptoms, anamneses related to autonomic nervous system function

**Symptoms**	Orthostatic hypotension, neurocardiogenic syncope, hypertensive crisis, tachycardia (at rest, during exercise or orthostasis), atrial fibrillation, long QT syndrome, heat/cold intolerance with skin color change
**Anamneses**	Radiation therapy for head and neck cancer, resection of neck tumors (bilateral), carotid endarterectomy, congestive heart failure, multiple system atrophy, diabetic neuropathy, Parkinson disease, long-term bedrest
**Total**	**☐Normal ☐Abnormal**

The anamneses and preoperative examinations related to myocardial injury

**Anamneses**	Coronary artery disease, myocardial injury, myocardial infarction, heart failure
**Preoperative examinations**	Serum cardiac troponin, serum myocardial enzyme, ST-T changes from intraoperative ECG, ultrasonic cardiogram, coronary angiogram
**Total**	**☐Normal ☐Abnormal**

The APAIS (the first four items)

**Item**	**Score**				
	1	2	3	4	5
1.I am worried about the anesthetic					
2.The anesthetic is on my mindcontinually					
3.I would like to know as much aspossible about the anesthetic					
4.Iam worried about the procedure					
**Total scores**					
l: Not at all, 2: Somewhat, 3: Moderate, 4: Moderately high, 5: Extremely					

The FRAIL scale

Item	Question	Score
1.fatigue	How much of the time during the past 4 weeks did you feel tired？<br>A=All or most B=Some, a little or none	□A=0<br>□B=1
2.resistance	In the past 4 weeks, by yourself and not using aids, do you have any difficulty walking up 10 steps without resting?	□Yes=1<br>□No=0
3.aerobic	In the past 4 weeks, by yourself and not using aids, do you have any difficulty walking 300 meters OR one block?	□Yes=1<br>□No=0
4.illness	Did your doctor ever tell you that you have?<br>Hypertension; Diabetes; Cancer (not a minor skin cancer)<br>Chronic lung disease; Heart attack; Congestive heart failure; Angina; Asthma; Arthritis; Kidney disease	□0-4 answers=0<br>□5-11 answers=1
5.loss of weight	Have you lost more than 5kgor 5% of your body weight inthe past year?	□Yes =1<br>□No=0
**Total scores**	□ROBUST=0 □PRE-FRAIL=1-2 □FRAIL≥3	
