# Supplementary material for: Protocol for development and validation of a prediction model for post-induction hypotension in elderly patients undergoing non-cardiac surgery: a prospective cohort study
Source: BMJ Open. 2023 Sep 21;13(9):e074181. doi: 10.1136/bmjopen-2023-074181 (PMC10514608; doi:10.1136/bmjopen-2023-074181)
Supplement: Author's manuscript [file bmjopen-2023-074181.draft_revisions.pdf]

BMJ Open

BMJ Open is committed to open peer review. As part of this commitment we make the peer review history of every article we publish publicly available.

When an article is published we post the peer reviewers’ comments and the authors’ responses online. We also post the versions of the paper that were used during peer review. These are the versions that the peer review comments apply to.

The versions of the paper that follow are the versions that were submitted during the peer review process. They are not the versions of record or the final published versions. They should not be cited or distributed as the published version of this manuscript.

BMJ Open is an open access journal and the full, final, typeset and author-corrected version of record of the manuscript is available on our site with no access controls, subscription charges or pay-per-view fees (http://bmjopen.bmj.com).

If you have any questions on BMJ Open’s open peer review process please email info.bmjopen@bmj.com

Protocol for development and validation of a prediction model for post-induction hypotension in elderly patients undergoing noncardiac surgery: a prospective cohort study

Journal:	*BMJ Open*
Manuscript ID	bmjopen-2023-074181
Article Type:	Protocol
Date Submitted by the Author:	29-Mar-2023
Complete List of Authors:	Hu, Jing-hui ; First Affiliated Hospital of Soochow University, Anesthesiology<br>Xu, Ning; First Affiliated Hospital of Soochow University, Anesthesiology<br>Bian, Zhen; First Affiliated Hospital of Soochow University, Anesthesiology<br>Shi, Hai-jing ; First Affiliated Hospital of Soochow University, Anesthesiology<br>Ji, Fu-Hai; First Affiliated Hospital of Soochow University, Anesthesiology<br>Peng, Ke; Soochow University,
Keywords:	Aged, Adult anaesthesia < ANAESTHETICS, Blood Pressure, Risk Factors, Adult surgery < SURGERY

SCHOLARONE™
Manuscripts

Protocol for development and validation of a prediction model for post-induction hypotension in elderly patients undergoing noncardiac surgery: a prospective cohort study

Jing-hui Hu[1,2,†], Ning Xu[1,2,†], Zhen Bian[1,2], Hai-jing Shi[1,2], Fu-hai Ji[1,2], Ke Peng[1,2,*]

[1] Department of Anaesthesiology, First Affiliated Hospital of Soochow University, Suzhou, Jiangsu, China

[2] Institute of Anaesthesiology, Soochow University, Suzhou, Jiangsu, China

[†] These authors contributed equally to this work.

***Correspondence to:**

Ke Peng, MD, PhD

Acting Vice Chair for Research and Associate Professor

Department of Anaesthesiology, First Affiliated Hospital of Soochow University

188 Shizi Street, Suzhou, Jiangsu 215006, China

Tel: +86-512-67780056; E-mail: pengke0422@163.com

ORCID: 0000-0003-2879-1759

Word count:

1548

For peer review only - http://bmjopen.bmj.com/site/about/guidelines.xhtml

Abstract

Introduction: Post-induction hypotension (PIH) is a common event in elderly surgical patients and is associated with increased postoperative morbidity and mortality. This study aims to develop and validate a PIH prediction model for elderly patients undergoing elective noncardiac surgery to identify potential PIH in advance and help to take preventive measures.

Methods and analysis: A total of 938 elderly surgical patients (n=657 for development and internal validation, n=281 for external validation) will be continuously recruited at The First Affiliated Hospital of Soochow University in Suzhou, China. The main outcome is PIH during the first 15 minutes after anaesthesia induction or before skin incision (whichever occurs first). We select candidate predictors based on published literature, professional knowledge, and clinical expertise. For model development, we will use the least absolute shrinkage and selection operator regression analysis and multivariable logistic regression. For internal validation, we will apply the bootstrapping technique. After model development and internal validation, external validation will be conducted in patients recruited in another time period. We will utilize the discrimination, calibration and max-rescaled Brier score in the external validation cohort. Furthermore, the clinical utility of the prediction model will be assessed using the decision curve analysis, and the results will be presented in a nomogram.

Ethics and dissemination: Ethical approval was obtained from the Ethics Committee of the First Affiliated Hospital of Soochow University (Approval No. 2023-012). This PIH risk prediction model will be published in a peer-reviewed journal.

Trial registration number: Chinese Clinical Trial Register (www.chictr.org.cn, ChiCTR2200066201)

For peer review only - http://bmjopen.bmj.com/site/about/guidelines.xhtml

Strengths and Limitations of This Study

1. This is a prospective cohort study to develop and validate a post-induction hypotension (PIH) prediction model for elderly patients undergoing noncardiac surgery.
2. The candidate predictors are selected based on the most recent published literature and clinical expertise, with easy availability and convenient clinical application.
3. This study includes the development, internal and external validation of the PIH prediction model.
4. The study is limited to anaesthesia induction period, without postoperative outcomes such as complications and mortality.
5. This is a single-centre study, and multicentre cohort studies are warranted for further external validation.

For peer review only

For peer review only - http://bmjopen.bmj.com/site/about/guidelines.xhtml

INTRODUCTION

Hypotension is common among patients undergoing surgery and general anaesthesia. It is closely correlated with adverse perioperative outcomes, such as myocardial injury, myocardial infarction, acute kidney injury, and death.[1,2] Accumulating evidence suggested that even short-term hypotension is hazardous to patients during noncardiac surgery.[3,4] Approximately 50% of hypotension events occur between anaesthesia induction and surgical incision,[5,6] which is called post-induction hypotension (PIH). Elderly patients are often frail with comorbidities and low physiological reserve, making them more prone to perioperative hypotension. Compared with intraoperative hypotension related to multiple factors, PIH is mainly attributable to patients' preoperative conditions and anaesthetic medications, which can be preventable.

Advanced age, baseline blood pressure, preoperative comorbidities, and type and dose of anaesthetics are associated with PIH.[7,8] Preoperative volume status and sympathetic activity are also involved in PIH.[9,10] With further investigation, some PIH prediction models have been developed; however, they are mostly derived from retrospective studies with missing data and limited number of factors, and some studies included patients in a single type of surgery only.[11-14] In recent years, new predictors have been reported, such as inferior vena cava-related parameters[15-17] and perfusion index.[18] Nonetheless, requiring specific equipment or techniques makes those predictors often difficult to be applied in the clinical practice.

Therefore, we designed this prospective cohort study to develop a universal and convenient PIH prediction model for elderly patients undergoing elective noncardiac surgery. Using this model, we can identify potential hypotension events in advance and take preventive measures to improve anaesthesia management and patient outcomes.

For peer review only - http://bmjopen.bmj.com/site/about/guidelines.xhtml

METHODS

Study design

This single-centre prospective cohort study is conducted at the First Affiliated Hospital of Soochow University between December 2022 and August 2023. The protocol and results of this study will be reported following the transparent reporting of a multivariable prediction model for individual prognosis or diagnosis (TRIPOD) checklist (**supplemental file 1**).[19]

Study population

Inclusion criteria

1. Aged 65 or greater and both genders.
2. American Society of Anaesthesiologists (ASA) physical status classification I−III.
3. Undergoing elective noncardiac surgery under general anaesthesia with tracheal intubation.
4. The duration of the surgery is longer than 30 minutes.

Exclusion criteria

1. Patients who have severe cardiovascular and cerebrovascular diseases, severe liver, kidney or lung dysfunction.
2. Undergoing nerve blocks, spinal or epidural anaesthesia.
3. Pre-existing tracheal intubation, tracheotomy, or ≥2 attempts of intubations.
4. Refusal for participation.

For peer review only

Study outcome

The study outcome is PIH during the first 15 minutes after anaesthesia induction or before skin incision (whichever occurs first). Preoperative baseline blood pressure will be recorded when patients are comfortably seated in the ward. During anaesthesia induction, patients will be monitored using noninvasive cuff blood pressure at 1-min interval. Hypotension is defined as a 30% reduction in mean arterial pressure (MAP)

For peer review only - http://bmjopen.bmj.com/site/about/guidelines.xhtml

from the baseline or MAP ≤65 mmHg.

Data collection

Study plan

One day before surgery, an independent investigator will identify eligible patients and inform them about the procedures and study aims. During the study, patients can withdraw the consent at any time. If the surgery is cancelled or the exclusion criteria are met, the patient will be excluded. **Figure 1** shows the study flow chart.

Candidate predictors are selected based on published data,[14] professional knowledge, and clinical expertise. The following clinical variables will be recorded in the case report forms (CRFs) (**supplemental file 2**).

Preoperative potential predictors

1. Demographic data including age, gender, and body mass index.
2. ASA physical status.
3. Comorbidities including hypertension, diabetes mellitus, cardiac diseases and the age-adjusted Charlson Comorbidity Index (aCCI) score.
4. Vital signs in the surgical ward including MAP and heart rate (HR).
5. Preoperative medications including cardiovascular drugs, oral antidiabetics, insulin and lipid-lowering drugs.
6. Preoperative anxiety assessed by the first four items of the Amsterdam Preoperative Anxiety and Information Scale (APAIS).[20]
7. Preoperative frailty evaluated with the FRAIL scale.[21,22]
8. Preoperative volume of fluid infusion and fasting duration.

Intraoperative potential predictors

1. Vital signs including MAP and HR immediately before anaesthesia induction.
2. Dose of anaesthetics used for induction, including propofol, etomidate, ciprofol, esketamine, fentanyl, and sufentanil.

For peer review only - http://bmjopen.bmj.com/site/about/guidelines.xhtml

Sample size estimation

According to previous literature,[5,8] the incidence of PIH was approximately 35% for patients undergoing elective noncardiac surgery. Considering a required number of 10 events per variable, we plan to recruit at least 657 patients (approximately 70% of total patients) for model development. Another 281 patients (approximately 30% of total patients) will be included for model external validation. Therefore, the planned sample size will be set to at least 938 patients.

Missing data

We will use multiple imputations when a variable has ≤5% missing data, while variables with >5% missing data will be excluded from the analysis.

Statistical analysis

Descriptive analysis and comparisons

Continuous variables will be presented as mean ± standard deviation (SD) and analysed using the Student's-t test when data are normally distributed, or presented as medians with interquartile ranges (IQRs) and analysed using the Mann-Whitney U test when data are not normally distributed. Categorical and ordinal data will be presented as number (percentages) and analysed using the $\chi 2$ or Fisher's exact tests. The absolute standardized difference (ASD) will be used to compare the baseline data between the development and validation cohort, with ASD >0.14 (i.e., $1.96 \times \sqrt{(657 + 281) / (657 \times 281)}$ indicating imbalance.

Development of the prediction model

We will use the least absolute shrinkage and selection operator (LASSO) regression analysis for variable shrinkage and selection in the development of linear regression model. After centralization and normalization of variables using the 10 times K cross-validation in the LASSO regression analysis, the best lambda value will be selected.

For peer review only - http://bmjopen.bmj.com/site/about/guidelines.xhtml

Based on the optimal predictors derived from the LASSO analysis, we will use the multivariable logistic regression analysis to develop a prediction model.

Validation of the prediction model

The internal validation of the model will be performed using a bootstrapping method. A total of 500 samples will be generated and drawn randomly with replacement, and each sample includes the same number of patients in the development cohort. Moreover, the external validation will be conducted in the external validation cohort. The discrimination capability of the model will be assessed using the area under the receiver operating characteristic curve (AUROC). Calibration of the model will be assessed using the Hosmer-Lemeshow goodness-of-fit statistic. The max-rescaled Brier score will be used to test the overall accuracy of the model. The clinical utility of the prediction model will be assessed using the decision curve analysis, and the results will be illustrated in a nomogram.

Software

All data will be analysed using the R software (version 4.2.2, www.R-project.org/) and the SPSS software (version 26.0, IBM SPSS Inc., Chicago, USA).

For peer review only

DISCUSSION

In this prospective cohort study, we will establish a model to predict PIH occurrence in elderly patients who are scheduled for elective noncardiac surgery. Based on the main risk factors identified, the model will illustrate their effects on PIH. The effectiveness of the model will be verified via internal and external validations. To facilitate clinical application, we will visualize the prediction model in a nomogram. The incidence of PIH, different factors, and the comparison with the existing models will be discussed in detail according to the study results.

To date, there have been many studies on the risk factors of PIH. A prospective multicenter observational study analysed 661 adult subjects who underwent elective noncardiac surgery under general anaesthesia.[8] They found that the probability of PIH increased with age, type II diabetes mellitus, the severity of hypertension at the time of operating room arrival, and the dose of propofol. In their study, the blood pressure was measured at a 5-min interval, which may result in missing of hypotension events. In our study, we set the measurement interval at every 1 minute for a total duration of 15 minutes or before skin incision, which improves the ability to detect PIH. In addition, the baseline MAP values will be determined in the ward preoperatively to avoid possible impact by anxiety after entering the operating room.[23] Although several PIH prediction models incorporating machine learning have been reported,[11-13] their data were retrospectively collected with a limited number of patients.

For peer review only

ETHICS AND DISSEMINATION

The study protocol was approved by the Ethics Committee of the First Affiliated Hospital of Soochow University on November 8, 2022, and then registered at the Chinese Clinical Trial Registry (http://www.chictr.org.cn, identifier: ChiCTR2200066201) on November 28, 2022. The second version of study protocol was approved by the Ethics Committee (Approval No. 2023-012) on January 10, 2023 and updated at the Chinese Clinical Trial Registry on January 28, 2023. All patients will provide their written informed consent to participate in this study. All acquired data will be deidentified, stored electronically and password protected. The final prediction model will be published via a peer-reviewed journal and presented at academic meetings.

For peer review only

For peer review only - http://bmjopen.bmj.com/site/about/guidelines.xhtml

Competing interests

None declared.

Funding

This study is supported by the Jiangsu Medical Association Anaesthesia Research Project (SYH-32021-0036 to KP), Suzhou Medical Health Science and Technology Innovation Project (SKY2022136 to KP), and National Natural Science Foundation of China (82072130 to FHJ).

Author contributions

HJH, NX, ZB, HJS, FHJ and KP participated in the study design. HJH, NX, ZB and HJS drafted the manuscript. FHJ and KP critically revised the manuscript. HJH and NX contributed to sample size calculation and statistic plan. HJH, NX and ZB developed the case report forms. KP is the primary investigator. KP and FHJ will acquire the research funding.

Acknowledgements

The authors thank Shuang-jie Wu, Zhou-lin Lu, and Li Zhou for their helpful collaboration in the Data Monitoring Committee.

Patient and public involvement

Patients and/or the public were not involved in the design, or conduct, or reporting, or dissemination plans of this research.

Patient consent for publication

Consent obtained directly from patient(s).

For peer review only - http://bmjopen.bmj.com/site/about/guidelines.xhtml

References

1. Walsh M, Devereaux PJ, Garg AX, et al. Relationship between intraoperative mean arterial pressure and clinical outcomes after noncardiac surgery: toward an empirical definition of hypotension. *Anesthesiology.* 2013;119(3):507-515.
2. Saugel B, Sessler DI. Perioperative Blood Pressure Management. *Anesthesiology.* 2021;134(2):250-261.
3. Sessler DI, Bloomstone JA, Aronson S, et al. Perioperative Quality Initiative consensus statement on intraoperative blood pressure, risk and outcomes for elective surgery. *Br J Anaesth.* 2019;122(5):563-574.
4. Wesselink EM, Kappen TH, Torn HM, Slooter AJC, van Klei WA. Intraoperative hypotension and the risk of postoperative adverse outcomes: a systematic review. *Br J Anaesth.* 2018;121(4):706-721.
5. Maheshwari K, Turan A, Mao G, et al. The association of hypotension during non-cardiac surgery, before and after skin incision, with postoperative acute kidney injury: a retrospective cohort analysis. *Anaesthesia.* 2018;73(10):1223-1228.
6. Saugel B, Reese PC, Sessler DI, et al. Automated Ambulatory Blood Pressure Measurements and Intraoperative Hypotension in Patients Having Noncardiac Surgery with General Anesthesia: A Prospective Observational Study. *Anesthesiology.* 2019;131(1):74-83.
7. Chen B, Pang QY, An R, Liu HL. A systematic review of risk factors for postinduction hypotension in surgical patients undergoing general anesthesia. *Eur Rev Med Pharmacol Sci.* 2021;25(22):7044-7050.
8. Jor O, Maca J, Koutna J, et al. Hypotension after induction of general anesthesia: occurrence, risk factors, and therapy. A prospective multicentre observational study. *J Anesth.* 2018;32(5):673-680.
9. Lim M, Ellahee P. Recommendations for postinduction hypotension: are they supported by the evidence? *Anesth Analg.* 2006;102(5).
10. Wong GTC, Irwin MG. Post-induction hypotension: a fluid relationship? *Anaesthesia.* 2021;76(1):15-18.
11. Lee J, Woo J, Kang AR, et al. Comparative Analysis on Machine Learning and Deep Learning to Predict Post-Induction Hypotension. *Sensors (Basel).* 2020;20(16).
12. Kang AR, Lee J, Jung W, et al. Development of a prediction model for hypotension after induction of anesthesia using machine learning. *PLoS One.* 2020;15(4):e0231172.
13. Kendale S, Kulkarni P, Rosenberg AD, Wang J. Supervised Machine-learning Predictive Analytics for Prediction of Postinduction Hypotension. *Anesthesiology.* 2018;129(4):675-688.
14. Reich DL, Hossain S, Krol M, et al. Predictors of hypotension after induction of general anesthesia. *Anesth Analg.* 2005;101(3):622-628.
15. Zhang H, Gao H, Xiang Y, Li J. Maximum inferior vena cava diameter predicts post-induction hypotension in hypertensive patients undergoing non-cardiac surgery under general anesthesia: A prospective cohort study. *Front Cardiovasc Med.* 2022;9:958259.
16. Aissaoui Y, Jozwiak M, Bahi M, et al. Prediction of post-induction hypotension by point-of-care echocardiography: A prospective observational study. *Anaesth Crit Care Pain Med.* 2022;41(4):101090.
17. Zhang J, Critchley LAH. Inferior Vena Cava Ultrasonography before General Anesthesia Can

For peer review only - http://bmjopen.bmj.com/site/about/guidelines.xhtml

Predict Hypotension after Induction. *Anesthesiology.* 2016;124(3):580-589.

18. Min JY, Chang HJ, Chu SJ, Chung MY. The Perfusion Index of the Ear as a Predictor of Hypotension Following the Induction of Anesthesia in Patients with Hypertension: A Prospective Observational Study. *J Clin Med.* 2022;11(21).
19. Collins GS, Reitsma JB, Altman DG, Moons KGM. Transparent Reporting of a multivariable prediction model for Individual Prognosis Or Diagnosis (TRIPOD). *Ann Intern Med.* 2015;162(10):735-736.
20. Moerman N, van Dam FS, Muller MJ, Oosting H. The Amsterdam Preoperative Anxiety and Information Scale (APAIS). *Anesth Analg.* 1996;82(3):445-451.
21. Abellan van Kan G, Rolland Y, Bergman H, Morley JE, Kritchevsky SB, Vellas B. The I.A.N.A Task Force on frailty assessment of older people in clinical practice. *J Nutr Health Aging.* 2008;12(1):29-37.
22. Kojima G. Quick and Simple FRAIL Scale Predicts Incident Activities of Daily Living (ADL) and Instrumental ADL (IADL) Disabilities: A Systematic Review and Meta-analysis. *J Am Med Dir Assoc.* 2018;19(12):1063-1068.
23. Song Q, Li J, Jiang Z. Provisional Decision-Making for Perioperative Blood Pressure Management: A Narrative Review. *Oxid Med Cell Longev.* 2022;2022:5916040.

For peer review only

For peer review only - http://bmjopen.bmj.com/site/about/guidelines.xhtml

Legends

Figure 1. The study flow chart

Supporting information

Supplemental file 1. Study checklist

Supplemental file 2. Case report forms

For peer review only

For peer review only - http://bmjopen.bmj.com/site/about/guidelines.xhtml

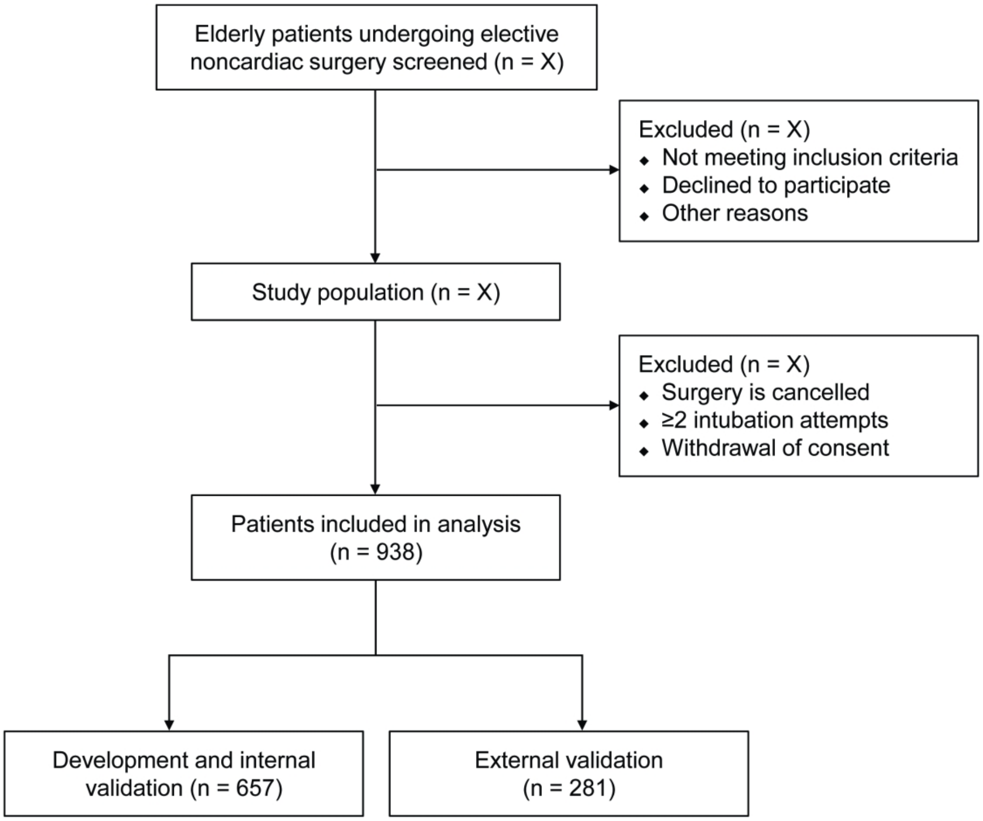

The study flow chart

213x177mm (300 x 300 DPI)

TRIPOD Checklist: Prediction Model Development and Validation

Section/Topic	Item		Checklist Item	Page
**Title and abstract**				
Title	1	D;V	Identify the study as developing and/or validating a multivariable prediction model, the target population, and the outcome to be predicted.	1
Abstract	2	D;V	Provide a summary of objectives, study design, setting, participants, sample size, predictors, outcome, statistical analysis, results, and conclusions.	2
**Introduction**				
Background and objectives	3a	D;V	Explain the medical context (including whether diagnostic or prognostic) and rationale for developing or validating the multivariable prediction model, including references to existing models.	4
	3b	D;V	Specify the objectives, including whether the study describes the development or validation of the model or both.	4
**Methods**				
Source of data	4a	D;V	Describe the study design or source of data (e.g., randomized trial, cohort, or registry data), separately for the development and validation data sets, if applicable.	5
	4b	D;V	Specify the key study dates, including start of accrual; end of accrual; and, if applicable, end of follow-up.	5
Participants	5a	D;V	Specify key elements of the study setting (e.g., primary care, secondary care, general population) including number and location of centres.	5
	5b	D;V	Describe eligibility criteria for participants.	5
	5c	D;V	Give details of treatments received, if relevant.	NA
Outcome	6a	D;V	Clearly define the outcome that is predicted by the prediction model, including how and when assessed.	5, 6
	6b	D;V	Report any actions to blind assessment of the outcome to be predicted.	NA
Predictors	7a	D;V	Clearly define all predictors used in developing or validating the multivariable prediction model, including how and when they were measured.	6
	7b	D;V	Report any actions to blind assessment of predictors for the outcome and other predictors.	NA
Sample size	8	D;V	Explain how the study size was arrived at.	7
Missing data	9	D;V	Describe how missing data were handled (e.g., complete-case analysis, single imputation, multiple imputation) with details of any imputation method.	7
Statistical analysis methods	10a	D	Describe how predictors were handled in the analyses.	7, 8
	10b	D	Specify type of model, all model-building procedures (including any predictor selection), and method for internal validation.	7, 8
	10c	V	For validation, describe how the predictions were calculated.	8
	10d	D;V	Specify all measures used to assess model performance and, if relevant, to compare multiple models.	8
	10e	V	Describe any model updating (e.g., recalibration) arising from the validation, if done.	NA
Risk groups	11	D;V	Provide details on how risk groups were created, if done.	NA
Development vs. validation	12	V	For validation, identify any differences from the development data in setting, eligibility criteria, outcome, and predictors.	NA
**Results**				
Participants	13a	D;V	Describe the flow of participants through the study, including the number of participants with and without the outcome and, if applicable, a summary of the follow-up time. A diagram may be helpful.	NA
	13b	D;V	Describe the characteristics of the participants (basic demographics, clinical features, available predictors), including the number of participants with missing data for predictors and outcome.	NA
	13c	V	For validation, show a comparison with the development data of the distribution of important variables (demographics, predictors and outcome).	NA
Model development	14a	D	Specify the number of participants and outcome events in each analysis.	NA
	14b	D	If done, report the unadjusted association between each candidate predictor and outcome.	NA
Model specification	15a	D	Present the full prediction model to allow predictions for individuals (i.e., all regression coefficients, and model intercept or baseline survival at a given time point).	NA
	15b	D	Explain how to the use the prediction model.	NA
Model performance	16	D;V	Report performance measures (with CIs) for the prediction model.	NA
Model-updating	17	V	If done, report the results from any model updating (i.e., model specification, model performance).	NA
**Discussion**				
Limitations	18	D;V	Discuss any limitations of the study (such as nonrepresentative sample, few events per predictor, missing data).	3
Interpretation	19a	V	For validation, discuss the results with reference to performance in the development data, and any other validation data.	NA
	19b	D;V	Give an overall interpretation of the results, considering objectives, limitations, results from similar studies, and other relevant evidence.	NA
Implications	20	D;V	Discuss the potential clinical use of the model and implications for future research.	9
**Other information**				
Supplementary information	21	D;V	Provide information about the availability of supplementary resources, such as study protocol, Web calculator, and data sets.	11
Funding	22	D;V	Give the source of funding and the role of the funders for the present study.	11

*Items relevant only to the development of a prediction model are denoted by D, items relating solely to a validation of a prediction model are denoted by V, and items relating to both are denoted D;V. We recommend using the TRIPOD Checklist in conjunction with the TRIPOD Explanation and Elaboration document.

For peer review only - http://bmjopen.bmj.com/site/about/guidelines.xhtml

Case Report Form

Preoperative potential predictors	
Age	
Gender	□Male □Female
BMI	
ASA physical status	□I □II □III
Hypertension	□Yes □No
Diabetes mellitus	□Yes □No
Cardiac diseases	□Yes □No
The age-adjusted Charlson Comorbidity Index (aCCI)	
MAP in the ward	
HR in the ward	
Medications	□Alpha blockers □Beta blockers<br>□Ca channels blockers<br>□ACE inhibitors □Diuretics<br>□Nitrate drug □Insulin<br>□Peroral antidiabetics<br>□Lipid-lowering drugs
The APAIS score (the first four items)	
The FRAIL scale score	□0 □1 □2
The volume of fluid infusion on the day of surgery	
Fasting duration on the day of surgery	
**Intraoperative potential predictors**	
MAP before induction	
HR before induction	
Dose of propofol	
Dose of etomidate	
Dose of ciprofol	
Dose of esketamine	
Dose of fentanyl	
Dose of sufentanil	

For peer review only

Outcome				
**Time point**	**HR（bpm）**	**NIBP（mmHg）**	**MAP（mmHg）**	**PIH#**
Immediately after induction		/		□Yes □No
1min after induction		/		□Yes □No
2min after induction				□Yes □No
3min after induction				□Yes □No
4min after induction				□Yes □No
5min after induction				□Yes □No
6min after induction		/		□Yes □No
7min after induction		/		□Yes □No
8min after induction		/		□Yes □No
9min after induction				□Yes □No
10min after induction				□Yes □No
11min after induction				□Yes □No
12min after induction				□Yes □No
13min after induction				□Yes □No
14min after induction		/		□Yes □No
15min after induction				□Yes □No
Skin incision*		/		□Yes □No

* the first 15 minutes after anesthesia induction or before the skin incision (whichever occurred first)

#The hypotension is defined as a 30% reduction in mean arterial blood pressure (MAP) to the baseline or MAP ≤65 mmHg.

For peer review only

The age-adjusted Charlson Comorbidity Index

Weight	Comorbid Condition
**1**	Myocardial infarction, congestive heart failure, peripheral vascular disease, cerebral vascular disease, dementia, chronic obstructive pulmonary disease, connective tissue disease, ulcer disease, mild liver disease, diabetes
**2**	Hemiplegia, moderate/severe renal disease, diabetes with end-organ damage, any tumor, leukemia/lymphoma
**3**	Moderate/severe liver disease
**6**	Metastatic solid tumor， AIDS
**1**	For each decade over age 40 years, up to 4 points
**Totoal**	
AIDS=acquired immune deficiency syndrome.	

The APAIS (the first four items)

Item	Score				
	1	2	3	4	5
1.I am worried about the anesthetic					
2.The anesthetic is on my mindcontinually					
3.I would like to know as much aspossible about the anesthetic					
4.Iam worried about the procedure					
**Total scores**					
l: Not at all, 2: Somewhat, 3: Moderate, 4: Moderately high, 5: Extremely					

The FRAIL scale

Item	Question	Score
1.fatigue	How much of the time during the past 4 weeks did you feel tired?<br>A=All or most B=Some, a little or none	□A=0<br>□B=1
2.resistance	In the past 4 weeks, by yourself and not using aids, do you have any difficulty walking up 10 steps without resting?	□Yes=1<br>□No=0
3.aerobic	In the past 4 weeks, by yourself and not using aids, do you have any difficulty walking 300 meters OR one block?	□Yes=1<br>□No=0
4.illness	Did your doctor ever tell you that you have?<br>Hypertension; Diabetes; Cancer (not a minor skin cancer)<br>Chronic lung disease; Heart attack; Congestive heart failure; Angina; Asthma; Arthritis; Kidney disease	□0-4 answers=0<br>□5-11 answers=1
5.loss of weight	Have you lost more than 5kgor 5% of your body weight inthe past year?	□Yes =1<br>□No=0
**Total scores**	□ROBUST=0 □PRE-FRAIL=1-2 □FRAIL≥3	

For peer review only

BMJ Open

Protocol for development and validation of a prediction model for post-induction hypotension in elderly patients undergoing noncardiac surgery: a prospective cohort study

Journal:	*BMJ Open*
Manuscript ID	bmjopen-2023-074181.R1
Article Type:	Protocol
Date Submitted by the Author:	09-Aug-2023
Complete List of Authors:	Hu, Jing-hui ; First Affiliated Hospital of Soochow University, Anesthesiology<br>Xu, Ning; First Affiliated Hospital of Soochow University, Anesthesiology<br>Bian, Zhen; First Affiliated Hospital of Soochow University, Anesthesiology<br>Shi, Hai-jing ; First Affiliated Hospital of Soochow University, Anesthesiology<br>Ji, Fu-Hai; First Affiliated Hospital of Soochow University, Anesthesiology<br>Peng, Ke; Soochow University,
**Primary Subject Heading**:	Anaesthesia
Secondary Subject Heading:	Surgery
Keywords:	Aged, Adult anaesthesia < ANAESTHETICS, Blood Pressure, Risk Factors, Adult surgery < SURGERY

SCHOLARONE™
Manuscripts

Protocol for development and validation of a prediction model for post-induction hypotension in elderly patients undergoing noncardiac surgery: a prospective cohort study

Jing-hui Hu[1,2,†], Ning Xu[1,2,†], Zhen Bian[1,2], Hai-jing Shi[1,2], Fu-hai Ji[1,2], Ke Peng[1,2,*]

[1] Department of Anaesthesiology, First Affiliated Hospital of Soochow University, Suzhou, Jiangsu, China

[2] Institute of Anaesthesiology, Soochow University, Suzhou, Jiangsu, China

[†] These authors contributed equally to this work.

***Correspondence to:**

Ke Peng, MD, PhD

Acting Vice Chair for Research and Associate Professor

Department of Anaesthesiology, First Affiliated Hospital of Soochow University

188 Shizi Street, Suzhou, Jiangsu 215006, China

Tel: +86-512-67780056; E-mail: pengke0422@163.com

ORCID: 0000-0003-2879-1759

Word count:

1749

For peer review only - http://bmjopen.bmj.com/site/about/guidelines.xhtml

Abstract

Introduction: Post-induction hypotension (PIH) is a common event in elderly surgical patients and is associated with increased postoperative morbidity and mortality. This study aims to develop and validate a PIH prediction model for elderly patients undergoing elective noncardiac surgery to identify potential PIH in advance and help to take preventive measures.

Methods and analysis: A total of 938 elderly surgical patients (n=657 for development and internal validation, n=281 for temporal validation) will be continuously recruited at The First Affiliated Hospital of Soochow University in Suzhou, China. The main outcome is PIH during the first 15 minutes after anaesthesia induction or before skin incision (whichever occurs first). We select candidate predictors based on published literature, professional knowledge, and clinical expertise. For model development, we will use the least absolute shrinkage and selection operator regression analysis and multivariable logistic regression. For internal validation, we will apply the bootstrapping technique. After model development and internal validation, temporal validation will be conducted in patients recruited in another time period. We will utilize the discrimination, calibration and max-rescaled Brier score in the temporal validation cohort. Furthermore, the clinical utility of the prediction model will be assessed using the decision curve analysis, and the results will be presented in a nomogram and a web-based risk calculator.

Ethics and dissemination: Ethical approval was obtained from the Ethics Committee of the First Affiliated Hospital of Soochow University (Approval No. 2023-012). This PIH risk prediction model will be published in a peer-reviewed journal.

Trial registration number: Chinese Clinical Trial Register (www.chictr.org.cn, ChiCTR2200066201)

For peer review only

For peer review only - http://bmjopen.bmj.com/site/about/guidelines.xhtml

Strengths and Limitations of This Study

1. This is a prospective cohort study to develop and validate a post-induction hypotension (PIH) prediction model for elderly patients undergoing noncardiac surgery.
2. The candidate predictors are selected based on the most recent published literature and clinical expertise, with easy availability and convenient clinical application.
3. This study includes the development, internal and temporal validation of the PIH prediction model.
4. The study is limited to anaesthesia induction period, without postoperative outcomes such as complications and mortality.
5. This is a single-centre study, and multicentre cohort studies are warranted for further external validation.

For peer review only

For peer review only - http://bmjopen.bmj.com/site/about/guidelines.xhtml

INTRODUCTION

Hypotension is common among patients undergoing surgery and general anaesthesia. It is closely correlated with adverse perioperative outcomes, such as myocardial injury, myocardial infarction, acute kidney injury, and death [1, 2]. Accumulating evidence suggested that even short-term hypotension is hazardous to patients during noncardiac surgery [3, 4]. Approximately 50% of hypotension events occur between anaesthesia induction and surgical incision [5, 6], which is called post-induction hypotension (PIH). Elderly patients are often frail with comorbidities and low physiological reserve, making them more prone to perioperative hypotension. Compared with intraoperative hypotension related to multiple factors, PIH is mainly attributable to patients' preoperative conditions and anaesthetic medications, which can be predictable and preventable.

Advanced age, baseline blood pressure, preoperative comorbidities, and type and dose of anaesthetics are associated with PIH [7, 8]. Preoperative volume status and sympathetic activity are also involved in PIH [9, 10]. With further investigation, some PIH prediction models have been developed; however, they are mostly derived from retrospective studies with missing data and limited number of factors, and some studies included patients in a single type of surgery only [11-14]. In recent years, new predictors have been reported, such as inferior vena cava-related parameters [15-17] and perfusion index [18]. Nonetheless, requiring specific equipment or techniques makes those predictors often difficult to be applied in the clinical practice.

Therefore, we designed this prospective cohort study to develop a universal and convenient PIH prediction model for elderly patients undergoing elective noncardiac surgery. Using this model, we can identify potential hypotension events in advance and take preventive measures to improve anaesthesia management and patient outcomes.

For peer review only - http://bmjopen.bmj.com/site/about/guidelines.xhtml

METHODS

Study design

This single-centre prospective cohort study is conducted at the First Affiliated Hospital of Soochow University between December 2022 and August 2023. The protocol and results of this study will be reported following the transparent reporting of a multivariable prediction model for individual prognosis or diagnosis (TRIPOD) checklist (**supplemental file 1**) [19].

Study population

Inclusion criteria

1. Aged 65 or greater and both genders.
2. American Society of Anaesthesiologists (ASA) physical status classification I−III.
3. Undergoing elective noncardiac surgery under general anaesthesia with tracheal intubation.
4. The duration of the surgery is longer than 30 minutes.

Exclusion criteria

1. Patients who have severe cardiovascular and cerebrovascular diseases, severe liver, kidney or lung dysfunction.
2. Undergoing nerve blocks, spinal or epidural anaesthesia.
3. Pre-existing tracheal intubation, tracheotomy, or ≥2 attempts of intubations.
4. Refusal for participation.

Study outcome

The study outcome is PIH during the first 15 minutes after anaesthesia induction or before skin incision (whichever occurs first). Preoperative baseline blood pressure will be recorded when patients are comfortably seated in the ward. During anaesthesia induction, patients will be monitored using noninvasive cuff blood pressure at 1-min interval. Hypotension is defined as either a 30% reduction in mean arterial pressure

For peer review only

For peer review only - http://bmjopen.bmj.com/site/about/guidelines.xhtml

(MAP) from baseline or absolute MAP ≤65 mmHg. Dual definitions for diagnosis of hypotension (either a relative reduction in MAP or an absolute threshold of MAP) have been used in many recent studies [20-25].

Data collection

Study plan

One day before surgery, a research assistant will identify eligible patients and inform them about the procedures and study aims. During the study, patients can withdraw the consent at any time. If the surgery is cancelled or the exclusion criteria are met, the patient will be excluded. **Figure 1** shows the study flow chart.

Candidate predictors are selected based on published data [14], professional knowledge, and clinical expertise. The following clinical variables will be recorded in the case report forms (CRFs) (**supplemental file 2**). The research assistant will fill out the preoperative potential predictors section of the form during the preoperative visit, and document variables in the intraoperative potential predictors section and values of HR and NIBP in the outcome section during the case when looking at the monitor.

During the analysis phase, we will calculate MAP at each time point using the traditional formula (MAP = diastolic blood pressure + 0.33×pulse pressure), and we will calculate the relative threshold (30% MAP reduction from baseline). Based on these, we can determine the occurrence of PIH.

Preoperative potential predictors

A total of 15 preoperative potential predictors:

1. Demographic data including age, gender, and body mass index.

2. ASA physical status.

3.Comorbidities including hypertension, diabetes mellitus and the age-adjusted Charlson Comorbidity Index (aCCI) score.

4. Autonomic nervous system function (assessed by symptoms and anamneses) [26, 27].

5. Myocardial injury (assessed by anamneses and preoperative examinations).

For peer review only - http://bmjopen.bmj.com/site/about/guidelines.xhtml

6. Vital signs in the surgical ward including MAP and heart rate (HR).

7. Preoperative anxiety assessed by the first four items of the Amsterdam Preoperative Anxiety and Information Scale (APAIS) [28].

8. Preoperative frailty evaluated with the FRAIL scale [29, 30].

9. Preoperative volume of fluid infusion and fasting duration.

Intraoperative potential predictors

A total of 8 intraoperative potential predictors:

1. Vital signs including MAP and HR immediately before anaesthesia induction.

2. Dose of anaesthetics used for induction, including propofol, etomidate, ciprofol, esketamine, fentanyl, and sufentanil.

Sample size estimation

According to previous literature [5, 8], the incidence of PIH was approximately 35% for patients undergoing elective noncardiac surgery. Considering a required number of 10 events per variable [31-33], we plan to recruit 657 patients for model development and internal validation (657=23×10/0.35). Another 281 patients will be included continuously for model temporal validation. Therefore, the planned sample size will be set to 938 patients.

Missing data

We will use multiple imputations when a variable has ≤5% missing data, while variables with >5% missing data will be excluded from the analysis.

Statistical analysis

Descriptive analysis and comparisons

Continuous variables will be presented as mean ± standard deviation (SD) and analysed using the Student's-t test when data are normally distributed, or presented as medians with interquartile ranges (IQRs) and analysed using the Mann-Whitney U test when

For peer review only - http://bmjopen.bmj.com/site/about/guidelines.xhtml

data are not normally distributed. Categorical and ordinal data will be presented as number (percentages) and analysed using the $\chi2$ or Fisher's exact tests. The absolute standardized difference (ASD) will be used to compare the baseline data between the development and validation cohort, with ASD >0.14 (i.e., 1.96 × $\sqrt{(657 + 281) / (657 \times 281)}$ indicating imbalance.

Development of the prediction model

The development cohort (n=657) will be used to construct the model. We will use the least absolute shrinkage and selection operator (LASSO) regression analysis for variable shrinkage and selection in the development of linear regression model. For centralization and normalization of variables, we will use the 10-fold cross-validation in the LASSO regression analysis, which will be repeated 10 times. In the 10 times 10-fold cross-validation, we will divide the development cohort into 10 equal parts. Then 9/10th of the data will be used to retrain the model and the remaining 1/10th will be used for validating the statistical accuracy in each time. The best lambda value will be selected. Based on the optimal predictors derived from the LASSO analysis, we will use the multivariable logistic regression analysis to develop a prediction model.

Validation of the prediction model

The internal validation of the model will be performed using a bootstrapping method in the development cohort (n=657). A total of 500 samples will be generated and drawn randomly with replacement, and each sample includes the same number of patients in the development cohort. Moreover, the temporal validation will be conducted in the temporal validation cohort (n=281). The discrimination capability of the model will be assessed using the area under the receiver operating characteristic curve (AUROC). Calibration of the model will be assessed using the Hosmer-Lemeshow goodness-of-fit statistic. The max-rescaled Brier score will be used to test the overall accuracy of the model. The clinical utility of the prediction model will be assessed using the decision curve analysis, and the results will be illustrated in a nomogram. Moreover, we will

For peer review only - http://bmjopen.bmj.com/site/about/guidelines.xhtml

develop a web-based risk calculator by the shiny package to provide practical guide for clinic.

Software

All data will be analysed using the R software (version 4.2.2, www.R-project.org/) and the SPSS software (version 26.0, IBM SPSS Inc., Chicago, USA).

Patient and public involvement

Patients and/or the public were not involved in the design, or conduct, or reporting, or dissemination plans of this research.

For peer review only

For peer review only - http://bmjopen.bmj.com/site/about/guidelines.xhtml

DISCUSSION

In this prospective cohort study, we will establish a model to predict PIH occurrence in elderly patients who are scheduled for elective noncardiac surgery. Based on the main risk factors identified, the model will illustrate their effects on PIH. The effectiveness of the model will be verified via internal and external validations. To facilitate clinical application, we will visualize the prediction model in a nomogram and a web-based risk calculator. The incidence of PIH, different factors, and the comparison with the existing models will be discussed in detail according to the study results.

To date, there have been many studies on the risk factors of PIH. A prospective multicenter observational study analysed 661 adult subjects who underwent elective noncardiac surgery under general anaesthesia [8]. They found that the probability of PIH increased with age, type II diabetes mellitus, the severity of hypertension at the time of operating room arrival, and the dose of propofol. In their study, the blood pressure was measured at a 5-min interval, which may result in missing of hypotension events. In our study, we set the measurement interval at every 1 minute for a total duration of 15 minutes or before skin incision, which improves the ability to detect PIH. In addition, the baseline MAP values will be determined in the ward preoperatively to avoid possible impact by anxiety after entering the operating room [34]. Although several PIH prediction models incorporating machine learning have been reported [11-13], their data were retrospectively collected with a limited number of patients.

In contrast, our study is based on a prospective cohort design. We select the candidate predictors based on published data, professional knowledge, and clinical expertise, which ensures the feasibility and practicality in finalizing the model. Regarding the APAIS score and FRAIL score, we will collect these two variables using the easy-to-use evaluation forms with good clinical practicality. Last, we will perform 10-fold cross-validation in the LASSO regression analysis and repeat it 10 times. According to the best lambda value, the optimal predictors will be derived from the LASSO analysis [35].

For peer review only - http://bmjopen.bmj.com/site/about/guidelines.xhtml

ETHICS AND DISSEMINATION

The study protocol was approved by the Ethics Committee of the First Affiliated Hospital of Soochow University on November 8, 2022, and then registered at the Chinese Clinical Trial Registry (http://www.chictr.org.cn, identifier: ChiCTR2200066201) on November 28, 2022. The second version of study protocol was approved by the Ethics Committee (Approval No. 2023-012) on January 10, 2023 and updated at the Chinese Clinical Trial Registry on January 28, 2023. All patients will provide their written informed consent to participate in this study. All acquired data will be deidentified, stored electronically and password protected. The final prediction model will be published via a peer-reviewed journal and presented at academic meetings.

For peer review only

For peer review only - http://bmjopen.bmj.com/site/about/guidelines.xhtml

Competing interests

None declared.

Funding

This study is supported by the College Students' Extracurricular Academic Research Project of Soochow University (KY2023101A to JHH), Jiangsu Medical Association Anaesthesia Research Project (SYH-32021-0036 (2021031) to KP), Suzhou Medical Health Science and Technology Innovation Project (SKY2022136 to KP), and National Natural Science Foundation of China (82072130 to FHJ).

Author contributions

JHH, NX, ZB, HJS, FHJ and KP participated in the study design. JHH, NX, ZB and HJS drafted the manuscript. FHJ and KP critically revised the manuscript. JHH and NX contributed to sample size calculation and statistic plan. JHH, NX and ZB developed the case report forms. KP is the primary investigator. JHH, KP and FHJ will acquire the research funding.

Acknowledgements

The authors thank Shuang-jie Wu, Zhou-lin Lu, and Li Zhou for their helpful collaboration in the Data Monitoring Committee.

Patient consent for publication

Consent obtained directly from patient(s).

For peer review only - http://bmjopen.bmj.com/site/about/guidelines.xhtml

References

1. Walsh M, Devereaux PJ, Garg AX, Kurz A, Turan A, Rodseth RN, Cywinski J, Thabane L, Sessler DI: **Relationship between intraoperative mean arterial pressure and clinical outcomes after noncardiac surgery: toward an empirical definition of hypotension**. *Anesthesiology* 2013, **119**(3):507-515.
2. Saugel B, Sessler DI: **Perioperative Blood Pressure Management**. *Anesthesiology* 2021, **134**(2):250-261.
3. Sessler DI, Bloomstone JA, Aronson S, Berry C, Gan TJ, Kellum JA, Plumb J, Mythen MG, Grocott MPW, Edwards MR *et al*: **Perioperative Quality Initiative consensus statement on intraoperative blood pressure, risk and outcomes for elective surgery**. *Br J Anaesth* 2019, **122**(5):563-574.
4. Wesselink EM, Kappen TH, Torn HM, Slooter AJC, van Klei WA: **Intraoperative hypotension and the risk of postoperative adverse outcomes: a systematic review**. *Br J Anaesth* 2018, **121**(4):706-721.
5. Maheshwari K, Turan A, Mao G, Yang D, Niazi AK, Agarwal D, Sessler DI, Kurz A: **The association of hypotension during non-cardiac surgery, before and after skin incision, with postoperative acute kidney injury: a retrospective cohort analysis**. *Anaesthesia* 2018, **73**(10):1223-1228.
6. Saugel B, Reese PC, Sessler DI, Burfeindt C, Nicklas JY, Pinnschmidt HO, Reuter DA, Südfeld S: **Automated Ambulatory Blood Pressure Measurements and Intraoperative Hypotension in Patients Having Noncardiac Surgery with General Anesthesia: A Prospective Observational Study**. *Anesthesiology* 2019, **131**(1):74-83.
7. Chen B, Pang QY, An R, Liu HL: **A systematic review of risk factors for postinduction hypotension in surgical patients undergoing general anesthesia**. *Eur Rev Med Pharmacol Sci* 2021, **25**(22):7044-7050.
8. Jor O, Maca J, Koutna J, Gemrotova M, Vymazal T, Litschmannova M, Sevcik P, Reimer P, Mikulova V, Trlicova M *et al*: **Hypotension after induction of general anesthesia: occurrence, risk factors, and therapy. A prospective multicentre observational study**. *J Anesth* 2018, **32**(5):673-680.
9. Lim M, Ellahee P: **Recommendations for postinduction hypotension: are they supported by the evidence?** *Anesth Analg* 2006, **102**(5).
10. Wong GTC, Irwin MG: **Post-induction hypotension: a fluid relationship?** *Anaesthesia* 2021, **76**(1):15-18.
11. Lee J, Woo J, Kang AR, Jeong Y-S, Jung W, Lee M, Kim SH: **Comparative Analysis on Machine Learning and Deep Learning to Predict Post-Induction Hypotension**. *Sensors (Basel)* 2020, **20**(16).
12. Kang AR, Lee J, Jung W, Lee M, Park SY, Woo J, Kim SH: **Development of a prediction model for hypotension after induction of anesthesia using machine learning**. *PLoS One* 2020, **15**(4):e0231172.
13. Kendale S, Kulkarni P, Rosenberg AD, Wang J: **Supervised Machine-learning Predictive Analytics for Prediction of Postinduction Hypotension**. *Anesthesiology* 2018, **129**(4):675-688.
14. Reich DL, Hossain S, Krol M, Baez B, Patel P, Bernstein A, Bodian CA: **Predictors of**

For peer review only

hypotension after induction of general anesthesia. *Anesth Analg* 2005, **101**(3):622-628.

15. Zhang H, Gao H, Xiang Y, Li J: **Maximum inferior vena cava diameter predicts post-induction hypotension in hypertensive patients undergoing non-cardiac surgery under general anesthesia: A prospective cohort study**. *Front Cardiovasc Med* 2022, **9**:958259.
16. Aissaoui Y, Jozwiak M, Bahi M, Belhadj A, Alaoui H, Qamous Y, Serghini I, Seddiki R: **Prediction of post-induction hypotension by point-of-care echocardiography: A prospective observational study**. *Anaesth Crit Care Pain Med* 2022, **41**(4):101090.
17. Zhang J, Critchley LAH: **Inferior Vena Cava Ultrasonography before General Anesthesia Can Predict Hypotension after Induction**. *Anesthesiology* 2016, **124**(3):580-589.
18. Min JY, Chang HJ, Chu SJ, Chung MY: **The Perfusion Index of the Ear as a Predictor of Hypotension Following the Induction of Anesthesia in Patients with Hypertension: A Prospective Observational Study**. *J Clin Med* 2022, **11**(21).
19. Collins GS, Reitsma JB, Altman DG, Moons KGM: **Transparent Reporting of a multivariable prediction model for Individual Prognosis Or Diagnosis (TRIPOD)**. *Ann Intern Med* 2015, **162**(10):735-736.
20. Khan AI, Fischer M, Pedoto AC, Seier K, Tan KS, Dalbagni G, Donat SM, Arslan-Carlon V: **The impact of fluid optimisation before induction of anaesthesia on hypotension after induction**. *Anaesthesia* 2020, **75**(5):634-641.
21. Shan XS, Hu LK, Wang Y, Liu HY, Chen J, Meng XW, Pu JX, Huang YH, Hou JQ, Feng XM *et al*: **Effect of Perioperative Dexmedetomidine on Delayed Graft Function Following a Donation-After-Cardiac-Death Kidney Transplant: A Randomized Clinical Trial**. *JAMA Netw Open* 2022, **5**(6):e2215217.
22. Yang YF, Zhu YJ, Long YQ, Liu HY, Shan XS, Feng XM, Peng K, Ji FH: **Withholding vs. Continuing Angiotensin-Converting Enzyme Inhibitors or Angiotensin Receptor Blockers Before Non-cardiac Surgery in Older Patients: Study Protocol for a Multicenter Randomized Controlled Trial**. *Front Med (Lausanne)* 2021, **8**:654700.
23. Shan XS, Dai HR, Zhao D, Yang BW, Feng XM, Liu H, Peng K, Ji FH: **Dexmedetomidine reduces acute kidney injury after endovascular aortic repair of Stanford type B aortic dissection: A randomized, double-blind, placebo-controlled pilot study**. *J Clin Anesth* 2021, **75**:110498.
24. Wang D, Long YQ, Sun Y, Zhu YJ, Feng XM, Liu H, Ji FH, Peng K: **Opioid-free total intravenous anesthesia for thyroid and parathyroid surgery: Protocol for a randomized, double-blind, controlled trial**. *Front Med (Lausanne)* 2022, **9**:939098.
25. Long YQ, Feng CD, Ding YY, Feng XM, Liu H, Ji FH, Peng K: **Esketamine as an Adjuvant to Ciprofol or Propofol Sedation for Same-Day Bidirectional Endoscopy: Protocol for a Randomized, Double-Blind, Controlled Trial With Factorial Design**. *Front Pharmacol* 2022, **13**:821691.
26. Goldberger JJ, Arora R, Buckley U, Shivkumar K: **Autonomic Nervous System Dysfunction: JACC Focus Seminar**. *J Am Coll Cardiol* 2019, **73**(10):1189-1206.
27. Ziemssen T, Siepmann T: **The Investigation of the Cardiovascular and Sudomotor Autonomic Nervous System-A Review**. *Front Neurol* 2019, **10**:53.
28. Moerman N, van Dam FS, Muller MJ, Oosting H: **The Amsterdam Preoperative Anxiety and Information Scale (APAIS)**. *Anesth Analg* 1996, **82**(3):445-451.
29. Abellan van Kan G, Rolland Y, Bergman H, Morley JE, Kritchevsky SB, Vellas B: **The I.A.N.A**

For peer review only - http://bmjopen.bmj.com/site/about/guidelines.xhtml

Task Force on frailty assessment of older people in clinical practice. *J Nutr Health Aging* 2008, **12**(1):29-37.

30. Kojima G: **Quick and Simple FRAIL Scale Predicts Incident Activities of Daily Living (ADL) and Instrumental ADL (IADL) Disabilities: A Systematic Review and Meta-analysis**. *J Am Med Dir Assoc* 2018, **19**(12):1063-1068.
31. Peduzzi P, Concato J, Feinstein AR, Holford TR: **Importance of events per independent variable in proportional hazards regression analysis. II. Accuracy and precision of regression estimates**. *J Clin Epidemiol* 1995, **48**(12):1503-1510.
32. Peduzzi P, Concato J, Kemper E, Holford TR, Feinstein AR: **A simulation study of the number of events per variable in logistic regression analysis**. *J Clin Epidemiol* 1996, **49**(12):1373-1379.
33. Moons KG, de Groot JA, Bouwmeester W, Vergouwe Y, Mallett S, Altman DG, Reitsma JB, Collins GS: **Critical appraisal and data extraction for systematic reviews of prediction modelling studies: the CHARMS checklist**. *PLoS Med* 2014, **11**(10):e1001744.
34. Song Q, Li J, Jiang Z: **Provisional Decision-Making for Perioperative Blood Pressure Management: A Narrative Review**. *Oxid Med Cell Longev* 2022, **2022**:5916040.
35. Bloniarz A, Liu H, Zhang CH, Sekhon JS, Yu B: **Lasso adjustments of treatment effect estimates in randomized experiments**. *Proc Natl Acad Sci U S A* 2016, **113**(27):7383-7390.

For peer review only

For peer review only - http://bmjopen.bmj.com/site/about/guidelines.xhtml

Legends

Figure 1. Study flow chart

Supporting information

Supplemental file 1. Study checklist

Supplemental file 2. Case report forms

For peer review only

For peer review only - http://bmjopen.bmj.com/site/about/guidelines.xhtml

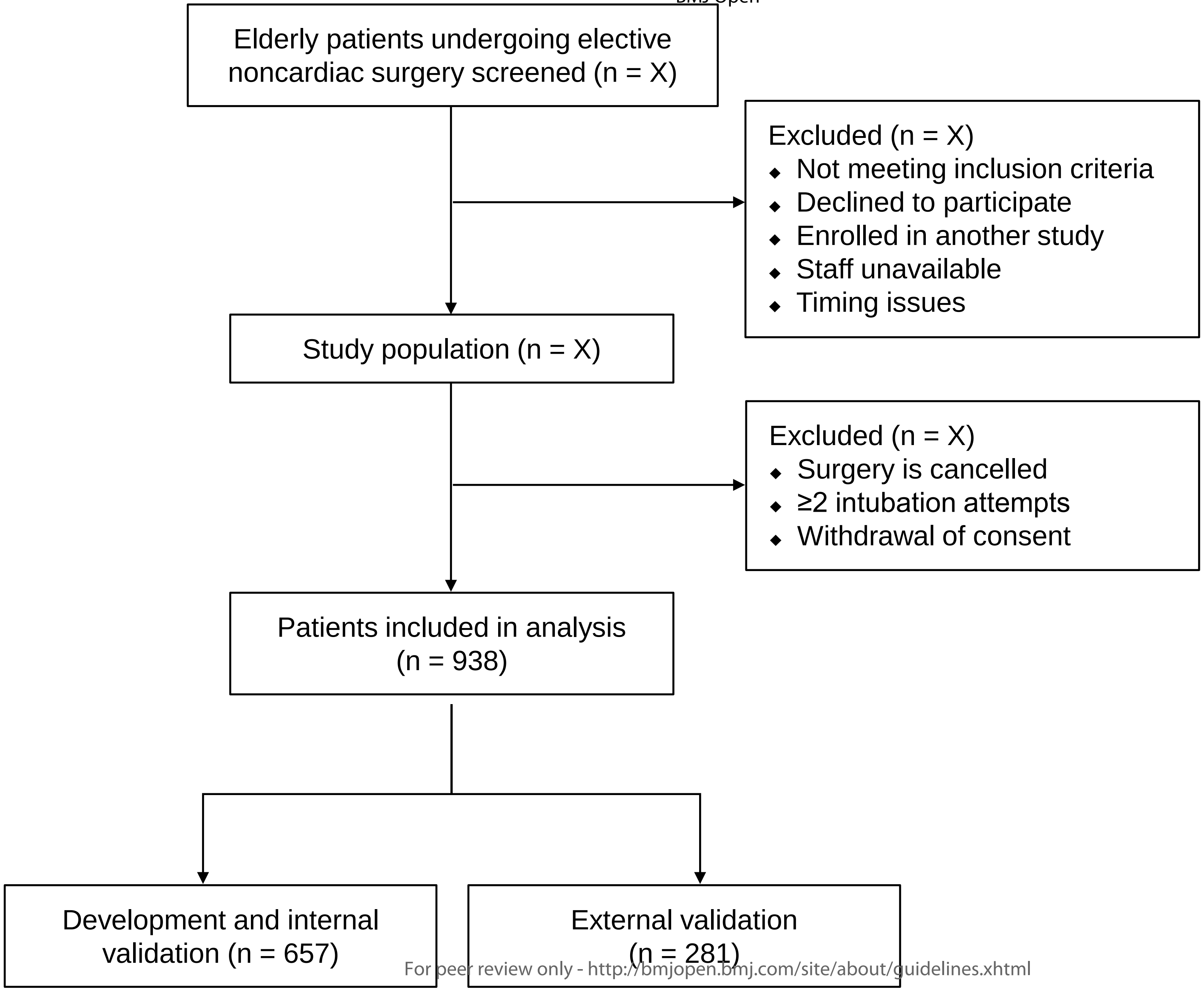
Elderly patients undergoing elective noncardiac surgery screened (n = X)
Excluded (n = X)
Not meeting inclusion criteria
Declined to participate
Enrolled in another study
Staff unavailable
Timing issues
Study population (n = X)
Excluded (n = X)
Surgery is cancelled
≥2 intubation attempts
Withdrawal of consent
Patients included in analysis (n = 938)
Development and internal validation (n = 657)
External validation (n = 281)

TRIPOD Checklist: Prediction Model Development and Validation

Section/Topic	Item		Checklist Item	Page
**Title and abstract**				
Title	1	D;V	Identify the study as developing and/or validating a multivariable prediction model, the target population, and the outcome to be predicted.	1
Abstract	2	D;V	Provide a summary of objectives, study design, setting, participants, sample size, predictors, outcome, statistical analysis, results, and conclusions.	2
**Introduction**				
Background and objectives	3a	D;V	Explain the medical context (including whether diagnostic or prognostic) and rationale for developing or validating the multivariable prediction model, including references to existing models.	4
	3b	D;V	Specify the objectives, including whether the study describes the development or validation of the model or both.	4
**Methods**				
Source of data	4a	D;V	Describe the study design or source of data (e.g., randomized trial, cohort, or registry data), separately for the development and validation data sets, if applicable.	5
	4b	D;V	Specify the key study dates, including start of accrual; end of accrual; and, if applicable, end of follow-up.	5
Participants	5a	D;V	Specify key elements of the study setting (e.g., primary care, secondary care, general population) including number and location of centres.	5
	5b	D;V	Describe eligibility criteria for participants.	5
	5c	D;V	Give details of treatments received, if relevant.	NA
Outcome	6a	D;V	Clearly define the outcome that is predicted by the prediction model, including how and when assessed.	5, 6
	6b	D;V	Report any actions to blind assessment of the outcome to be predicted.	NA
Predictors	7a	D;V	Clearly define all predictors used in developing or validating the multivariable prediction model, including how and when they were measured.	6, 7
	7b	D;V	Report any actions to blind assessment of predictors for the outcome and other predictors.	NA
Sample size	8	D;V	Explain how the study size was arrived at.	7
Missing data	9	D;V	Describe how missing data were handled (e.g., complete-case analysis, single imputation, multiple imputation) with details of any imputation method.	7
Statistical analysis methods	10a	D	Describe how predictors were handled in the analyses.	7, 8
	10b	D	Specify type of model, all model-building procedures (including any predictor selection), and method for internal validation.	8
	10c	V	For validation, describe how the predictions were calculated.	8
	10d	D;V	Specify all measures used to assess model performance and, if relevant, to compare multiple models.	8
	10e	V	Describe any model updating (e.g., recalibration) arising from the validation, if done.	NA
Risk groups	11	D;V	Provide details on how risk groups were created, if done.	NA
Development vs. validation	12	V	For validation, identify any differences from the development data in setting, eligibility criteria, outcome, and predictors.	NA
**Results**				
Participants	13a	D;V	Describe the flow of participants through the study, including the number of participants with and without the outcome and, if applicable, a summary of the follow-up time. A diagram may be helpful.	NA
	13b	D;V	Describe the characteristics of the participants (basic demographics, clinical features, available predictors), including the number of participants with missing data for predictors and outcome.	NA
	13c	V	For validation, show a comparison with the development data of the distribution of important variables (demographics, predictors and outcome).	NA
Model development	14a	D	Specify the number of participants and outcome events in each analysis.	NA
	14b	D	If done, report the unadjusted association between each candidate predictor and outcome.	NA
Model specification	15a	D	Present the full prediction model to allow predictions for individuals (i.e., all regression coefficients, and model intercept or baseline survival at a given time point).	NA
	15b	D	Explain how to the use the prediction model.	NA
Model performance	16	D;V	Report performance measures (with CIs) for the prediction model.	NA
Model-updating	17	V	If done, report the results from any model updating (i.e., model specification, model performance).	NA
**Discussion**				
Limitations	18	D;V	Discuss any limitations of the study (such as nonrepresentative sample, few events per predictor, missing data).	3
Interpretation	19a	V	For validation, discuss the results with reference to performance in the development data, and any other validation data.	NA
	19b	D;V	Give an overall interpretation of the results, considering objectives, limitations, results from similar studies, and other relevant evidence.	NA
Implications	20	D;V	Discuss the potential clinical use of the model and implications for future research.	10
**Other information**				
Supplementary information	21	D;V	Provide information about the availability of supplementary resources, such as study protocol, Web calculator, and data sets.	NA
Funding	22	D;V	Give the source of funding and the role of the funders for the present study.	12

*Items relevant only to the development of a prediction model are denoted by D, items relating solely to a validation of a prediction model are denoted by V, and items relating to both are denoted D;V. We recommend using the TRIPOD Checklist in conjunction with the TRIPOD Explanation and Elaboration document.

For peer review only - http://bmjopen.bmj.com/site/about/guidelines.xhtml

Case Report Form

Preoperative potential predictors (15)	
Age	
Gender	□Male □Female
BMI	
ASA physical status	□I □II □III
Hypertension	□Yes □No
Diabetes mellitus	□Yes □No
The age-adjusted Charlson Comorbidity Index (aCCI)	
Autonomic nervous system function	□Normal □Abnormal
Cardiac function	□Normal □Abnormal
NIBP in the ward	/ (MAP:)
HR in the ward	
The APAIS score (the first four items)	
The FRAIL scale score	□0 □1 □2
The volume of fluid infusion on the day of surgery	
Fasting duration on the day of surgery	
**Intraoperative potential predictors (8)**	
NIBP before induction	/ (MAP:)
HR before induction	
Dose of propofol	
Dose of etomidate	
Dose of ciprofol	
Dose of esketamine	
Dose of fentanyl	
Dose of sufentanil	

For peer review only

30% reduction in MAP from baseline threshold: ______________

**Outcome**				
**Time point**	**HR（bpm）**	**NIBP（mmHg）**	**MAP（mmHg）**	**PIH#**
Immediately after induction		/		□Yes □No
1min after induction		/		□Yes □No
2min after induction		/		□Yes □No
3min after induction		/		□Yes □No
4min after induction		/		□Yes □No
5min after induction		/		□Yes □No
6min after induction		/		□Yes □No
7min after induction		/		□Yes □No
8min after induction		/		□Yes □No
9min after induction		/		□Yes □No
10min after induction		/		□Yes □No
11min after induction		/		□Yes □No
12min after induction		/		□Yes □No
13min after induction		/		□Yes □No
14min after induction		/		□Yes □No
15min after induction		/		□Yes □No
Skin incision*		/		□Yes □No

* The first 15 minutes after anaesthesia induction or before skin incision (whichever occurred first)

# Hypotension is defined as a 30% reduction in MAP from baseline or MAP ≤65 mmHg.

For peer review only

The age-adjusted Charlson Comorbidity Index

Weight	Comorbid Condition
**1**	Myocardial infarction, congestive heart failure, peripheral vascular disease, cerebral vascular disease, dementia, chronic obstructive pulmonary disease, connective tissue disease, ulcer disease, mild liver disease, diabetes
**2**	Hemiplegia, moderate/severe renal disease, diabetes with end-organ damage, any tumor, leukemia/lymphoma
**3**	Moderate/severe liver disease
**6**	Metastatic solid tumor， AIDS
**1**	For each decade over age 40 years, up to 4 points
**Total**	
AIDS=acquired immune deficiency syndrome.	

The symptoms, anamneses related to autonomic nervous system function

**Symptoms**	Orthostatic hypotension, neurocardiogenic syncope, hypertensive crisis, tachycardia (at rest, during exercise or orthostasis), atrial fibrillation, long QT syndrome, heat/cold intolerance with skin color change
**Anamneses**	Radiation therapy for head and neck cancer, resection of neck tumors (bilateral), carotid endarterectomy, congestive heart failure, multiple system atrophy, diabetic neuropathy, Parkinson disease, long-term bedrest
**Total**	☐**Normal** ☐**Abnormal**

The anamneses and preoperative examinations related to myocardial injury

**Anamneses**	Coronary artery disease, myocardial injury, myocardial infarction, heart failure
**Preoperative examinations**	Serum cardiac troponin, serum myocardial enzyme, ST-T changes from intraoperative ECG, ultrasonic cardiogram, coronary angiogram
**Total**	☐**Normal** ☐**Abnormal**

The APAIS (the first four items)

**Item**	**Score**				
	1	2	3	4	5
1.I am worried about the anesthetic					
2.The anesthetic is on my mindcontinually					
3.I would like to know as much aspossible about the anesthetic					
4.Iam worried about the procedure					
**Total scores**					
l: Not at all, 2: Somewhat, 3: Moderate, 4: Moderately high, 5: Extremely					

For peer review only

The FRAIL scale

Item	Question	Score
1.fatigue	How much of the time during the past 4 weeks did you feel tired?<br>A=All or most B=Some, a little or none	□A=0<br>□B=1
2.resistance	In the past 4 weeks, by yourself and not using aids, do you have any difficulty walking up 10 steps without resting?	□Yes=1<br>□No=0
3.aerobic	In the past 4 weeks, by yourself and not using aids, do you have any difficulty walking 300 meters OR one block?	□Yes=1<br>□No=0
4.illness	Did your doctor ever tell you that you have?<br>Hypertension; Diabetes; Cancer (not a minor skin cancer) Chronic lung disease; Heart attack; Congestive heart failure; Angina; Asthma; Arthritis; Kidney disease	□0-4 answers=0<br>□5-11 answers=1
5.loss of weight	Have you lost more than 5kgor 5% of your body weight inthe past year?	□Yes =1<br>□No=0
**Total scores**	□ROBUST=0 □PRE-FRAIL=1-2 □FRAIL≥3	

For peer review only
